# Non-Physician Areas in Japan: Typological Classification Based on Regional Demographic Characteristics

**DOI:** 10.2196/101570

**Published:** 2026-10-09

**Authors:** Takashi Kuwayama, Kazuhiko Kotani

**Affiliations:** 1 Center for Community Medicine, Jichi Medical University, 3311-1 Yakushiji, Shimotsuke-City, Tochigi, 3290498, Japan, +81-285-58-7394

**Keywords:** non-physician areas, rural health care, regional characteristics, cluster, machine learning

## Abstract

**Background:**

Across Japan, there are “non-physician areas” where medical institutions are not present and health care access is difficult. Although these areas are defined as a single administrative category, they may vary according to regional demographic characteristics. Understanding this possible heterogeneity may be important for developing more context-sensitive strategies to improve health care accessibility.

**Objective:**

This study examined whether non-physician areas can be classified based on regional demographic characteristics using an unsupervised machine learning model.

**Methods:**

A total of 590 non-physician areas were identified from the national survey conducted by the Ministry of Health, Labour and Welfare of Japan (2019). Data on regional demographic characteristics were also obtained. After *z* score standardization, k-means clustering was used for classification. The optimal number of clusters was determined using silhouette scores. The data on non-physician areas were divided into training (354/590, 60.0% areas) and validation (236/590, 40.0% areas) datasets. The reproducibility of the cluster structure was evaluated using Jensen-Shannon distance and chi-square tests, and principal component analysis (PCA) was used to examine the overall structure of the clusters.

**Results:**

The optimal number of clusters was 3 (silhouette score=0.266), indicating that non-physician areas could be classified into 3 types. The cluster distributions of the training and validation datasets were consistent (Jensen-Shannon distance=0.017; *P*=.76), supporting the reproducibility of the cluster structure. The PCA suggested that differences among non-physician areas could be interpreted along 2 axes, including population age structure and settlement size. The 3 clusters represented younger, intermediate, and older types, characterized by differences in demographic and settlement characteristics.

**Conclusions:**

The present study demonstrated that administratively defined non-physician areas in Japan are not uniform and can be classified into 3 demographically distinct types. The typology may provide a framework for understanding differences not captured by the current administrative designation and for considering more context-sensitive health care strategies.

## Introduction

Geographic barriers to health care remain an important challenge in rural areas, where limited availability of medical institutions and professionals can result in disparities in access to care [1]. Health care accessibility is increasingly recognized as a multidimensional concept that extends beyond the simple presence or absence of health care facilities [2]. Demographic structure, settlement patterns, transportation, and local social environments interact to shape access to care, particularly in rural areas [1-3]. Therefore, ensuring equitable access to health care in rural areas remains an important issue for health care policy [1,2].

Definitions of rural areas vary across countries and health systems [4]. Such areas may be identified according to geographic remoteness, population density, distance to or travel time to health care facilities, or shortages of health care professionals [4]. Consequently, the populations and health care challenges encompassed by these definitions are not necessarily uniform across settings [4]. Internationally, this diversity highlights the importance of understanding rural areas in relation to their demographic, geographic, and social contexts [4].

In Japan, there are geographically isolated areas where medical provision is difficult. Within these areas, there are “non-physician areas” [5]. Non-physician areas are defined as areas that, in principle, do not have medical institutions, where 50 or more people reside within an approximately 4 km radius centered on the main location of the area, and where access to medical institutions is difficult [5]. According to the 2019 national survey, there were 590 such areas [5]. Various efforts have been made to improve health care accessibility in rural areas in Japan, including policies to secure physicians for rural areas [6].

Non-physician areas themselves may be heterogeneous. They are found in locations ranging from areas near cities to mountains and islands and are considered to vary according to regional demographic characteristics [7,8]. Regional demographic characteristics refer to area-level demographic and sociodemographic attributes, such as population age, sex, and household structure [9-13]. Thus, even when the areas meet the same administrative definition of a non-physician area, their population structure, settlement characteristics, transportation conditions, and corresponding health care needs are presumed to differ. Despite this recognition, non-physician areas in Japan continue to be treated largely as a single administrative category, and little evidence exists regarding whether they differ according to demographic characteristics.

Understanding this heterogeneity may be important for developing effective strategies to reduce health care disparities and support more context-sensitive health care planning rather than applying uniform strategies to all non-physician areas. However, previous studies have not fully examined the demographic characteristics of non-physician areas [3,4]. Therefore, this study examined whether non-physician areas could be classified based on regional demographic characteristics. Developing a data-driven typology may provide a framework for better understanding non-physician areas and for considering health care strategies tailored to their different regional contexts.

## Methods

### Data

The present study did not involve human participant recruitment. All 590 government-defined non-physician areas listed in the 2019 National Survey of Non-physician Areas conducted by the Ministry of Health, Labour and Welfare of Japan were included, representing a nationwide census of eligible non-physician areas rather than a sampled population. Data on regional demographic characteristics, including population composition and household structure, were obtained from the same survey [14].

### Study Design

Unsupervised machine learning was applied to classify non-physician areas based on regional demographic characteristics. The dataset was randomly divided into training (354/590, 60.0% areas) and validation (236/590, 40.0% areas) datasets using a fixed random seed (random_state=42) to ensure reproducibility. The cluster structure was derived using the training data, and its reproducibility was evaluated using the validation data.

### Clustering Method

K-means clustering was used for this purpose. K-means is an unsupervised learning method that automatically groups data with similar characteristics based on multiple variables and assigns each data point to the nearest cluster center to minimize the within-cluster variance [15]. The variables used for k-means clustering were the proportions of the older population, young population, working-age population, older households, and male individuals, as well as the total population and the number of households. Automobile ownership was excluded from the analysis because more than half of the areas had missing data for this variable. For sex composition, only the proportion of male individuals was used to avoid redundancy, as the male and female proportions sum to 1. K-means clustering was performed with 10 initializations (n_init=10) using a fixed random seed (random_state=42). The number of clusters (k) was evaluated from k=2 to k=7 using silhouette scores, and the number of clusters corresponding to the highest score was selected [16]. The silhouette score is an index that evaluates how well each data point is grouped within its cluster and how well it is separated from other clusters; higher values indicate better clustering.

### Reproducibility

The k-means model fitted to the training data was applied to the validation data, and each validation area was assigned to the nearest cluster centroid. The cluster distributions between the training and validation datasets were compared. Reproducibility was evaluated using the Jensen-Shannon distance [17] and the chi-square test, using the training data as the expected distribution.

If no significant difference was observed in the chi-square test, it was interpreted that there was no statistical difference in the cluster distributions, and the cluster structure was considered reproducible.

### Standardization

All numerical variables were standardized using *z* scores (mean 0, SD 1). Population and household variables showed right-skewed distributions and were log-transformed prior to standardization. The mean *z* scores for each variable were calculated for each cluster to evaluate the cluster characteristics. Missing values for the total number of households (1/590, 0.2%) and the number of older households (63/590, 10.7%) were imputed using the median of each variable before calculation of the derived variables. Because the proportions of missing values for these variables were relatively limited, median imputation was used to retain all areas in the analysis. In contrast, automobile ownership was excluded because more than half of the areas had missing data, making extensive imputation inappropriate.

### Principal Component Analysis

Principal component analysis (PCA) was performed to examine the overall structure of the clusters and their relative positions [18]. PCA was conducted using the same 7 standardized variables used for k-means clustering, and the first and second principal components were visualized. For sex composition, only the proportion of male individuals was used to avoid redundancy, as the male and female proportions sum to 1. PCA loadings were calculated to evaluate the contributions of variables to each principal component.

### Statistical Analysis

Skewed variables were log-transformed. Comparisons between clusters were performed using 1-way ANOVA. When significant differences were observed, post hoc multiple comparisons with Bonferroni correction were conducted. Analyses were conducted using Python (version 3.13; Python Software Foundation) and *scikit-learn* (version 1.8). Statistical significance was set at 5.0%.

### Ethical Considerations

The present study was approved by the Jichi Medical University Research Ethics Committee (22-138). All data were obtained from publicly available sources and did not include any personal information.

## Results

The number of clusters was selected based on silhouette scores, and the optimal number was k=3. The silhouette scores were 0.254 (k=2), 0.266 (k=3), 0.204 (k=4), 0.203 (k=5), 0.210 (k=6), and 0.188 (k=7), with k=3 having the highest score. The number of areas assigned to each cluster was 173, 184, and 233, representing 29.3%, 31.2%, and 39.5% of the 590 areas, respectively (Table 1). The number of areas assigned to each cluster was 103, 108, and 143 in the training data, representing 29.1%, 30.5%, and 40.4% of the 354 training areas, respectively, and 70, 76, and 90 in the validation data, representing 29.7%, 32.2%, and 38.1% of the 236 validation areas, respectively.

**Table 1.** Characteristics of the 3 clusters in all non-physician areas (n=590).

Variables	All, median (IQR)	Cluster 1 (n=173), median (IQR)	Cluster 2 (n=184), median (IQR)	Cluster 3 (n=233), median (IQR)	Cluster 1 vs 2, *P* value^a^	Cluster 1 vs 3, *P* value^a^	Cluster 2 vs 3, *P* value^a^	Overall, *P* value^b^
Total households	59.0 (38.0–118.2)	39.0 (28.0–54.0)	160.0 (119.0–229.2)	50.0 (36.0–65.0)	<.001^c^	<.001	<.001	<.001
Older households	28.0 (19.0–48.0)	16.0 (9.0–25.0)	58.5 (28.0–94.0)	28.0 (22.0–40.0)	<.001	<.001	<.001	<.001
Total male individuals	61.0 (38.0–127.0)	48.0 (34.0–64.0)	176.5 (130.8–240.5)	43.0 (31.0–61.0)	<.001	.23	<.001	<.001
Male individuals aged 0-14 years	4.0 (1.0–9.0)	4.0 (3.0–7.0)	12.0 (7.0–18.5)	1.0 (0.0–3.0)	<.001	<.001	<.001	<.001
Male individuals aged 15-64 years	30.0 (18.0–62.0)	25.0 (18.0–34.0)	86.5 (64.0–126.5)	18.0 (12.0–26.0)	<.001	<.001	<.001	<.001
Male individuals aged 65-69 years	8.0 (4.0–15.0)	5.0 (3.0–8.0)	22.0 (15.0–30.0)	6.0 (4.0–8.0)	<.001	.008	<.001	<.001
Male individuals aged ≥70 years	22.0 (13.0–42.0)	12.0 (7.0–18.0)	52.5 (40.8–81.5)	17.0 (14.0–26.0)	<.001	<.001	<.001	<.001
Total female individuals	64.0 (41.0–134.0)	48.0 (32.0–64.0)	183.0 (136.8–261.0)	48.0 (36.0–68.0)	<.001	.14	<.001	<.001
Female individuals aged 0-14 years	4.0 (1.0–9.0)	4.0 (2.0–6.0)	11.0 (8.0–18.0)	1.0 (0.0–3.0)	<.001	<.001	<.001	<.001
Female individuals aged 15-64 years	25.0 (14.0–53.0)	21.0 (15.0–30.0)	76.0 (55.0–111.5)	14.0 (10.0–22.0)	<.001	<.001	<.001	<.001
Female individuals aged 65-69 years	6.0 (4.0–13.0)	4.0 (3.0–6.0)	18.0 (13.0–27.0)	5.0 (3.0–8.0)	<.001	.002	<.001	<.001
Female individuals aged ≥70 years	31.0 (19.0–59.0)	17.0 (12.0–24.0)	75.5 (55.8–111.2)	28.0 (21.0–37.0)	<.001	<.001	<.001	<.001
Total population	124.5 (77.0–262.8)	94.0 (68.0–128.0)	358.0 (267.0–498.5)	91.0 (67.0–129.0)	<.001	>.99	<.001	<.001
Population aged 0-14 years	7.0 (3.0–18.0)	8.0 (5.0–14.0)	22.0 (15.8–38.0)	3.0 (1.0–5.0)	<.001	<.001	<.001	<.001
Population aged 15-64 years	53.0 (31.0–112.0)	47.0 (33.0–65.0)	161.5 (119.2–242.8)	32.0 (23.0–46.0)	<.001	<.001	<.001	<.001
Population aged 65-69 years	14.0 (8.0–28.0)	10.0 (7.0–13.0)	39.0 (28.0–56.0)	11.0 (7.0–16.0)	<.001	<.001	<.001	<.001
Population aged ≥70 years	52.0 (33.0–101.0)	28.0 (20.0–41.0)	130.5 (97.0–196.2)	45.0 (35.0–64.0)	<.001	<.001	<.001	<.001

a *P* values were obtained using Bonferroni multiple comparisons.

b *P* values were obtained by a 1-way ANOVA.

c Values in italics indicate statistical significance (*P*<.05).

The Jensen-Shannon distance between the 2 distributions was 0.017, indicating consistency. The chi-square test yielded *χ*²_2_=0.546 (*P*=.76), indicating no significant difference. These results indicate that the cluster structure is reproducible.

PCA was performed for data visualization (Figure 1). The first principal component explained 44.4% of the variance, and the second explained 26.1%, with a cumulative contribution of approximately 70.5%.

**Figure 1.**
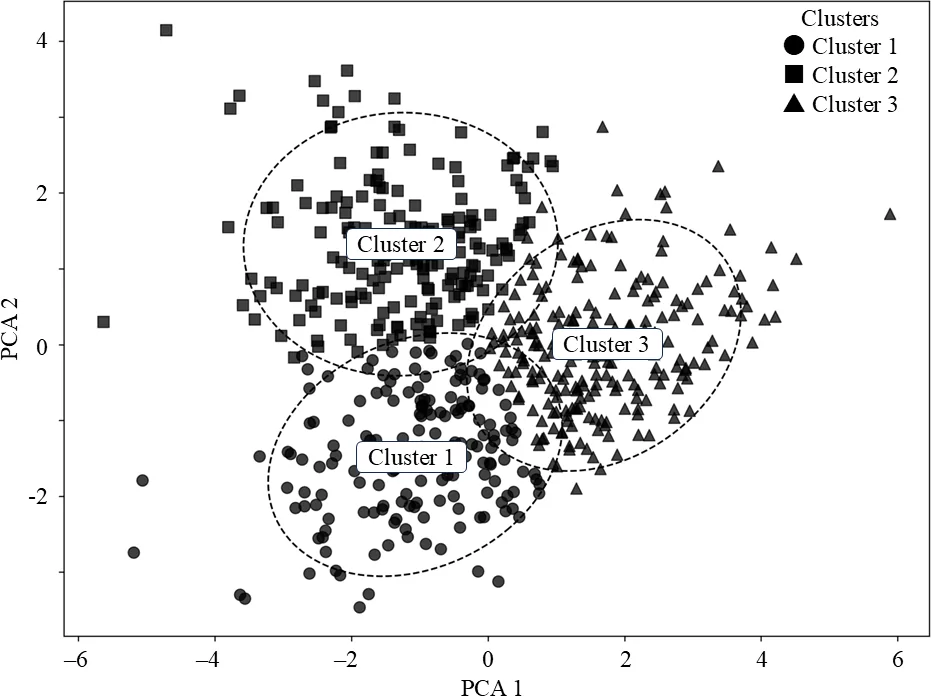
Principal component analysis (PCA) scatter plot of non-physician areas. The PCA scatter plot of non-physician areas is based on 7 standardized variables. Each point represents an individual area, and the marker shapes indicate the 3 clusters identified by unsupervised clustering. Dashed ellipses indicate the distribution ranges of each cluster.

In the PCA plot, the 3 clusters were distributed across different regions. Clusters 1 and 2 were located mainly in the negative region of PCA 1, whereas cluster 3 was located in the positive region. Along PCA 2, cluster 1 was located in the negative region, cluster 2 in the positive region, and cluster 3 in the intermediate region of the PCA plot.

PCA loadings showed that PCA 1 was positively associated with the proportion of the older population and older households and negatively associated with the working-age population and total population. PCA 2 was positively associated with the total population and number of households (Table 2). The mean *z* scores showed differences in population composition and household characteristics among the clusters (Table 3). Table 3 presents both the standardized values used for clustering and the corresponding descriptive statistics on the original scale.

**Table 2.** Principal component loadings for the principal component analysis (PCA).

Variables	PCA loadings 1	PCA loadings 2
Older population (%)	0.518	0.226
Young population (%)	–0.368	–0.211
Working-age population (%)	–0.488	–0.193
Older households (%)	0.389	–0.037
Male individuals (%)	–0.252	–0.241
Population (n)	–0.299	0.609
Households (n)	–0.232	0.66

**Table 3.** Profiles of the 3 clusters based on the training dataset (n=354)^a^.

Variables	Cluster 1 (n=103)		Cluster 2 (n=108)		Cluster 3 (n=143)	
	Median (IQR)	*z* score, mean (SD)	Median (IQR)	*z* score, mean (SD)	Median (IQR)	*z* score, mean (SD)
Older population (%)	44.2 (36.1-47.8)	–0.816 (0.681)	48.1 (42.8-52.9)	–0.355 (0.751)	61.4 (56.2-68.1)	0.856 (0.646)
Young population (%)	8.6 (6.6-12.1)	0.755 (1.021)	6.3 (4.7-8.2)	0.166 (0.801)	3.1 (1.3-5.0)	–0.669 (0.612)
Working-age population (%)	47.9 (44.6-52.9)	0.697 (0.726)	45.5 (41.6-49.2)	0.37 (0.810)	35.7 (29.2-39.3)	–0.782 (0.740)
Older households (%)	40.0 (24.6-47.5)	–0.362 (1.096)	40.6 (25.0-50.0)	–0.44 (0.818)	59.2 (52.3-70.9)	0.593 (0.728)
Male individuals (%)	50.6 (48.0-53.4)	0.562 (0.956)	48.9 (46.9-50.0)	0.055 (0.624)	46.6 (43.8-49.0)	–0.446 (1.054)
Population (n)	94.0 (69.0-131.5)	–0.54 (0.523)	358.0 (271.8-527.2)	1.243 (0.663)	91.0 (68.0-129.5)	–0.549 (0.525)
Households (n)	38.0 (29.0-53.5)	–0.699 (0.528)	164.5 (122.8-242.5)	1.22 (0.667)	48.0 (35.0-64.5)	–0.418 (0.544)

a Original-scale values are presented as median (IQR), and standardized values are presented as mean *z* scores. Population and number of households were log-transformed before standardization.

## Discussion

### Principal Findings

The principal contribution of the present study was not the statistical identification of 3 clusters itself but the demonstration that administratively defined non-physician areas could comprise multiple demographic contexts that might require different approaches. This study classified non-physician areas into 3 types, and the reproducibility of the classification was then confirmed. The PCA results suggested that the differences among areas could be interpreted along 2 axes, including population age structure and settlement size, which could represent the underlying structural differences in population composition and living environment.

On the basis of the mean *z* scores, cluster 1, characterized by a small settlement size and relatively higher proportions of younger and working-age populations, was named the “younger type.” Cluster 3, characterized by higher proportions of the older population and older households and a smaller population size, was named the “older type.” Cluster 2 showed an intermediate age structure and a relatively larger settlement size and was named the “intermediate type.” These results imply that non-physician areas are not uniform [7]. The analyzed typology therefore complements the existing administrative classification by providing an interpretable, data-driven framework for understanding demographic heterogeneity within areas that are currently treated as a single category.

### Comparison With Prior Work

Although the administrative designation of non-physician areas is unique to Japan, the heterogeneity observed among these areas is consistent with the broader international literature on rural areas [4,19]. A scoping review of 240 empirical studies from Western countries showed that medical deserts have been defined and characterized using diverse criteria, including population density, population size, distance to health professionals or facilities, and broader sociodemographic conditions [4]. That review suggests that rural areas must not necessarily be regarded as a single type of geographical disadvantage; rather, the health care response may depend on the demographic, geographic, and infrastructural context of each area [4,19,20].

The approach used in the present study might have broader relevance beyond Japan. Data-driven classification of rural areas according to their demographic characteristics may help identify meaningful subtypes within rural populations and thereby support more context-sensitive health care strategies. The potential international relevance of the present study therefore lies not in assuming that the 3 clusters identified in Japan are directly transferable to other countries but in demonstrating a data-driven approach for identifying heterogeneity within rural areas [4].

In Japan, efforts to improve health care accessibility in rural areas have included policies to secure and deploy physicians to rural areas [6]. The present study findings suggest that, in addition to such efforts to reduce the number of non-physician areas, understanding differences among the remaining areas may help inform the allocation of limited health care resources.

Viewed in this context, the 3 clusters identified in the present study may have different implications for health care resource allocation and workforce planning. Cluster 1, characterized by a relatively younger population and small settlement size, may represent communities in which a combination of regional primary care facilities, outreach services, and digitally supported care [19,21] could help maintain health care access. Cluster 2 had a relatively larger settlement size and an intermediate age structure, suggesting that comparatively greater population concentration could make periodic outreach services or mobile clinics more operationally feasible and could support the allocation of shared health care personnel across a wider catchment population. In contrast, cluster 3 was characterized by a smaller and markedly older population, suggesting that strategies requiring patients to travel to centralized services may be less suitable and that greater emphasis needs to be placed on locally accessible services, home-based care, and outreach by multidisciplinary health care teams. From a workforce perspective, these differences also suggest that a uniform strategy for physician deployment may be insufficient: larger settlements are more amenable to regular staffing or shared workforce models, whereas smaller and older communities may require flexible deployment strategies, including visiting professionals and mobile or home-based services [4].

Telemedicine may be 1 component of such place-sensitive strategies because it can potentially reduce geographic barriers and provide access to care without requiring patients or health care professionals to travel long distances [22]. Evidence from rural settings indicates clinical benefits; for example, a systematic review of telemental health in rural areas reported improvements in mental health symptoms [23]. However, telemedicine should not be assumed to be equally feasible across all non-physician areas. Its implementation depends on factors such as digital infrastructure, access to devices, digital literacy, and the suitability of remote consultation for the clinical condition [22]. In particular, communities with older populations may face greater challenges in using digitally delivered services, while some clinical needs still require physical examination or face-to-face care [22,24]. Therefore, telemedicine must be considered as a complement to, rather than a replacement for, in-person, outreach, and home-based services, with the appropriate combination depending on local circumstances [21].

Accordingly, the typology found in the present study may serve as a framework for considering place-sensitive combinations of health care workforce deployment, outreach services, transportation support, home-based care, and digitally supported care, rather than prescribing a single service model for all non-physician areas [19-21]. Such an approach is consistent with the broader primary health care principle of organizing health services around population needs and promoting equitable access to care [25]. The typology should therefore not be viewed as prescribing a specific intervention for each cluster but as a framework for identifying which additional local information and health care resources should be considered when designing strategies to improve health care accessibility.

These interpretations should be regarded as hypotheses for health care planning rather than direct conclusions of the present study. Future research should examine whether the identified cluster types are associated with differences in health care workforce availability, geographic accessibility, health care use, digital infrastructure, and health outcomes. Such studies could also evaluate whether particular models of service delivery, including mobile services, home-based care, and telemedicine, differ in feasibility or effectiveness across the analyzed cluster types.

### Limitations

This study has several limitations. First, k-means clustering assumes a linear structure. Second, the variables were limited to population and household characteristics. Important factors relevant to health care access and service planning, including health care workforce availability, health care use, digital infrastructure, transportation networks, and socioeconomic conditions, were not included. In particular, automobile ownership was excluded because of the high proportion of missing data. Therefore, the identified clusters do not capture differences in private vehicle access, which may be relevant to health care accessibility in rural areas. In addition, missing values for older households were imputed using the median. Although the proportion of missing values was relatively limited, this imputation may have reduced variation in this variable and may have influenced the cluster assignments. Third, the relationship between the proposed clusters and health outcomes has not been examined. Therefore, the typology should be considered a demographic framework rather than evidence that particular health care interventions are effective for specific clusters. Future studies linking the typology with health care resources, use, and outcomes are needed to clarify its practical utility.

Fourth, the typology should be interpreted within the framework of the Japanese administrative definition of non-physician areas. This definition excluded settlements with fewer than 50 residents and did not incorporate travel distance or travel time to the nearest medical institutions. Therefore, the identified clusters reflect demographic variations among administratively defined non-physician areas rather than the full spectrum of spatial health care accessibility. Future studies integrating geographic information system–based travel measures may provide a more comprehensive understanding of accessibility.

Finally, the analysis was based on the 2019 national survey, which was the most recent nationwide dataset available at the time of the study. The demographic characteristics of some non-physician areas may change over time. Consequently, the present cluster assignments should not be interpreted as permanent classifications but rather as a baseline typology based on the available national data. Future studies using updated survey data will be important to examine the temporal stability of the proposed typology.

### Conclusions

In the present study, administratively defined non-physician areas in Japan are not uniform but are demographically heterogeneous and can be classified into 3 types. The typology provides a framework for understanding differences that are not captured by the current administrative designation and suggests that different combinations of health care strategies may be appropriate across the 3 types. Rather than prescribing specific interventions for each cluster, the typology may highlight the potential for tailoring health care workforce deployment, outreach services, transportation support, home-based care, and digitally supported care to different regional needs and contexts. Although the specific clusters identified in Japan may not be directly transferable to other countries, the data-driven approach to identifying heterogeneity within rural areas may also have relevance to medical deserts and other rural settings internationally. Future studies incorporating geographic accessibility, health care resources, use, health outcomes, and updated national data are needed to evaluate the practical applicability and temporal stability of the findings.

## Abbreviations

PCA: principal component analysis
